# Rehabilitation of a deep bite patient with worn dentition using minimally invasive approach: A 3‐year follow‐up

**DOI:** 10.1002/ccr3.5121

**Published:** 2021-11-22

**Authors:** Mahya Hasanzade, Safoura Ghodsi, Negin Yaghoobi

**Affiliations:** ^1^ Dental Research Center Department of Prosthodontics Dentistry Research Institute Tehran University of Medical Sciences Tehran Iran

**Keywords:** bruxism, full mouth rehabilitation, occlusal onlay, tooth wear, vertical dimension loss

## Abstract

Full mouth rehabilitation of severely worn teeth represents a challenging situation for dental clinicians. This case report describes the minimally invasive interdisciplinary approach for treatment of severely worn dentition with a loss of vertical dimension of occlusion. After 3 years of follow‐up, no complication was observed.

## Graphical abstract

Rehabilitation of severely worn teeth in deep bite patient is considered as a challenging situation. Minimally invasive restorations and occlusal ceramic overlays are presented as a reliable and durable option in treatment of these cases.

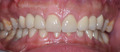

## 1 INTRODUCTION

Occlusal wear is a prevalent phenomenon that is defined as flattening of cusp tips or loss of incisal edges by physiological or pathological processes.^1^ Severe tooth wear may provoke some unfavorable effects including occlusal disharmony, decreased vertical dimension of occlusion (VDO), pulpitis or hypersensitivity related to dentine exposure, poor aesthetic, decrease in the masticatory function, and temporomandibular joint disorders.^2^, ^3^ Some authors believe that the original VDO could be preserved in some types of occlusal wear with compensatory mechanisms of dentoalveolar complex including extrusion of worn teeth and alveolar process.^1^, ^4^ However, increasing the VDO is indicated in some cases to provide sufficient restorative space, improve the esthetic, reduce steep anterior guidance, or eliminate occlusal interferences.^4^, ^5^

Intentional endodontic treatment, crown lengthening, and full coverage restorations have been suggested for restoring worn teeth with insufficient restorative space. However, these conventional procedures are invasive and destruct a noticeable amount of tooth structure.^6^ Minimally invasive restorations were introduced by advancement in adhesion and dental bonding agents to preserve the remaining tooth structure.^7^ Based on related literature, conservative approaches are more reasonable, while more aggressive procedures could be postponed until older ages.^6^, ^8^ Occlusal veneers are considered as a conservative method for increasing VDO in cases with severely worn dentition. Durability of these restorations and ease of fabrication, candidate them as suitable conservative treatment options.^6^, ^9^

This clinical report aims to describe full‐mouth rehabilitation of a patient with severe deep bite and worn dentition using occlusal ceramic overlays.

## 2 CASE PRESENTATION

**FIGURE 1:**
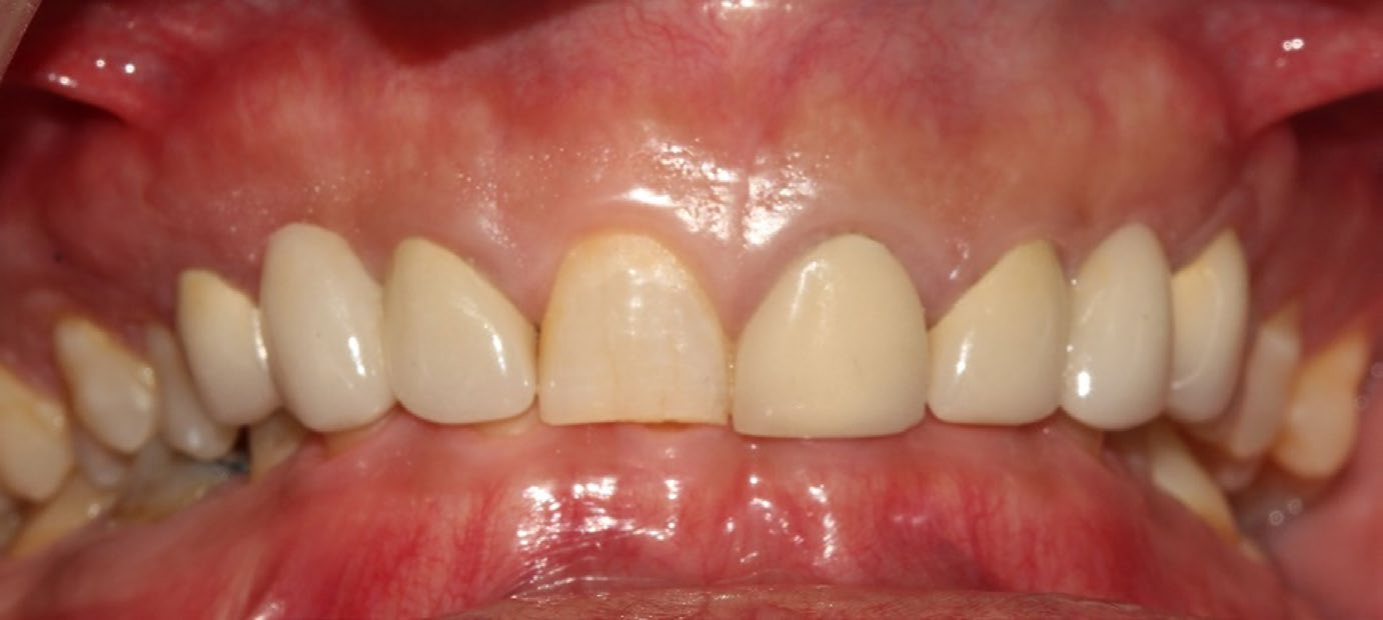
Intraoral frontal view

**FIGURE 2:**
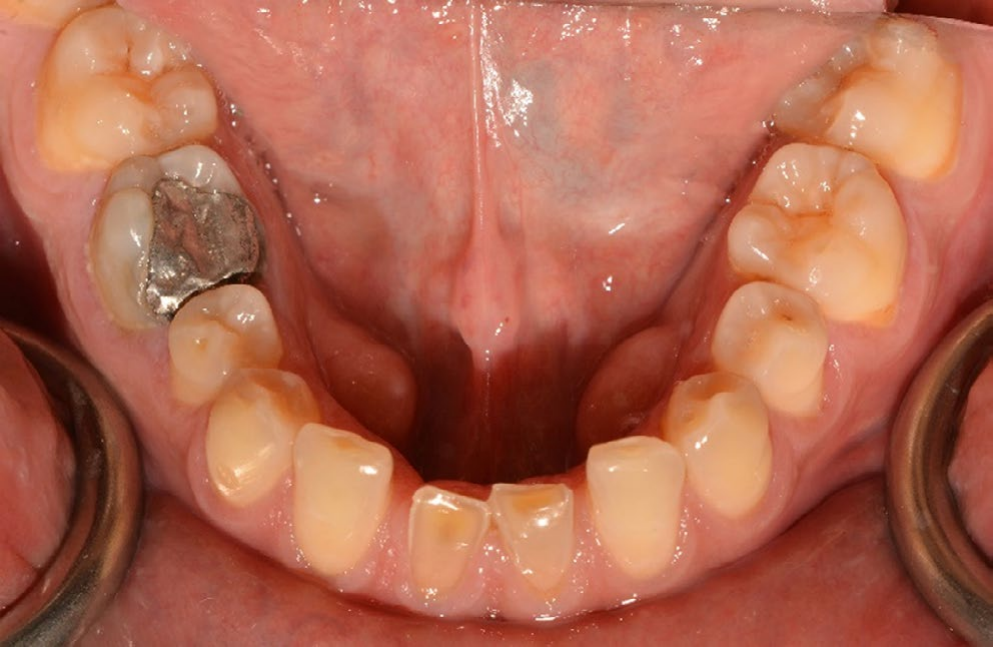
Mandibular occlusal view

**FIGURE 3:**
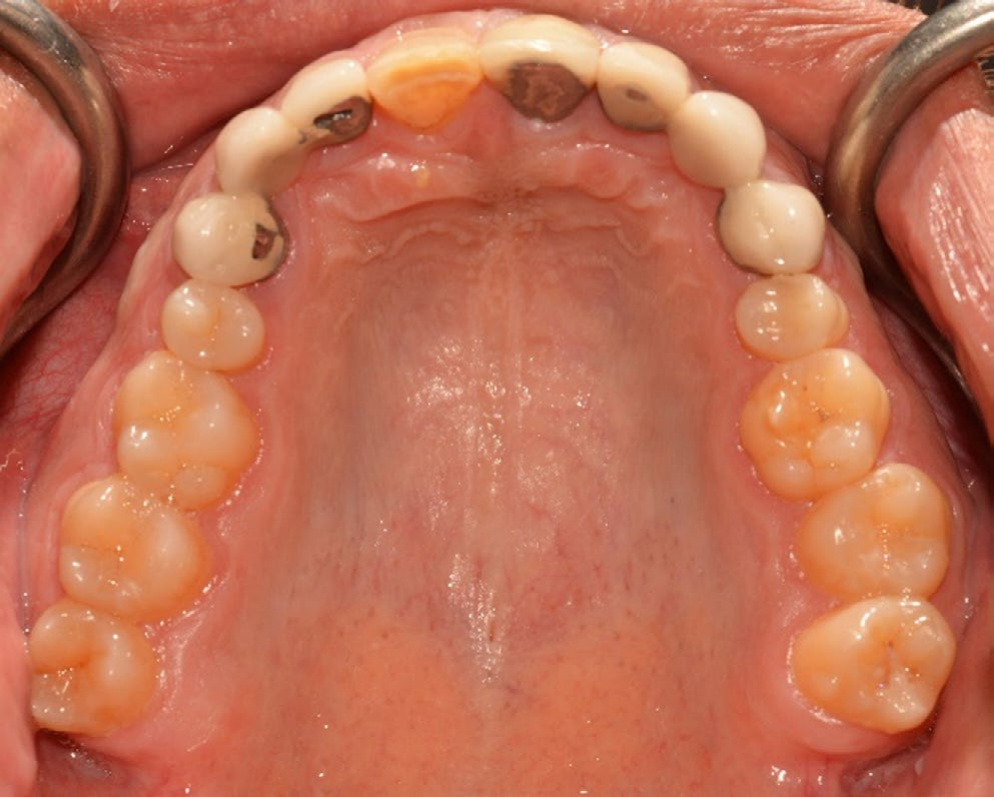
Maxillary occlusal view

**FIGURE 4:**
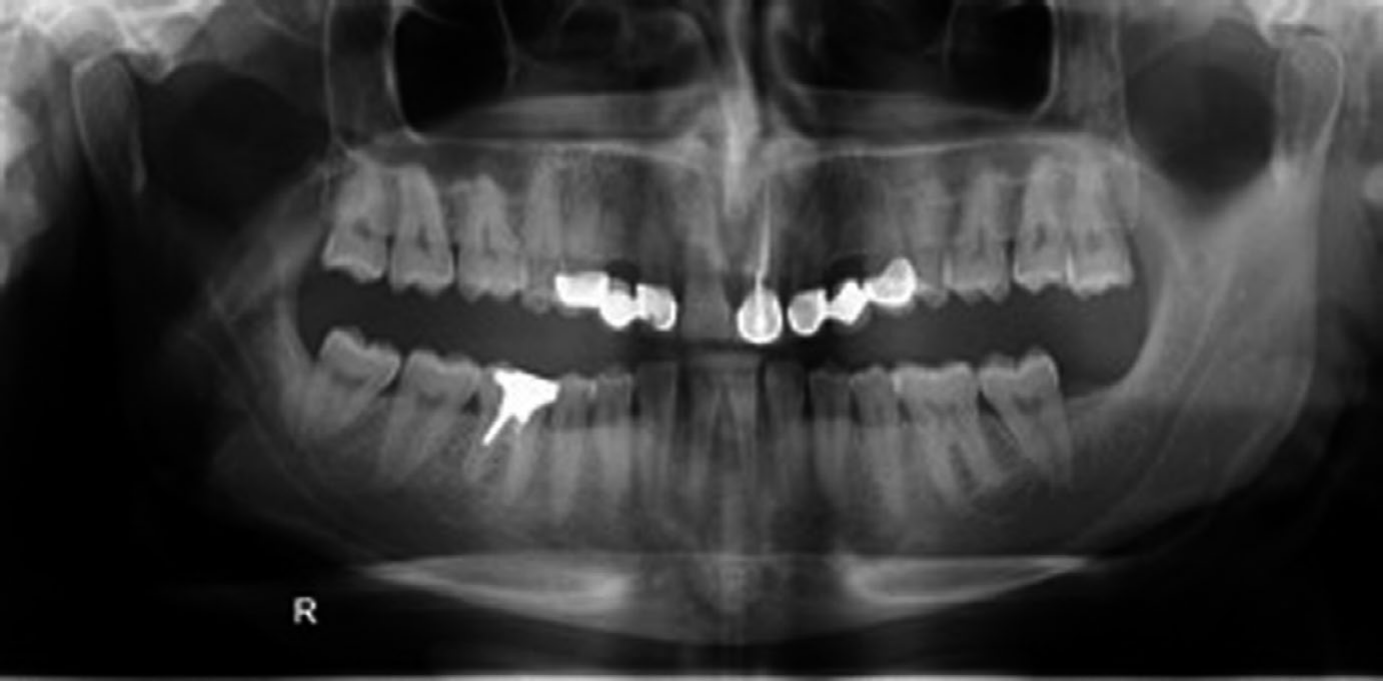
Initial panoramic view

A 34‐ year‐old man was referred to the department of prosthodontics for treatment of his severely worn dentition. His chief complaint was moderate pain in posterior gingival tissue of upper teeth related to tapping force of lower anterior teeth, and aesthetic and functional problems related to excessive wear in labial surface of lower incisors. Extra oral examination revealed a europrosopic face with slightly shortened lower facial height. No limitations or deviation was observed during maximum mouth opening. Palpation of muscles, lymph nodes, and TMJs confirmed normal conditions. However, deep overbite occlusion and midline discrepancy were noticeable in the frontal view (Figure 1). Intraoral examination indicated severe localized wear and exposure of dentin in lower incisors (Figure 2). Posterior occlusion revealed interferences on teeth number 16 and 17; due to the buccal inclination of this tooth, the extraction of maxillary third molar was considered before recording the centric relation. The other potential cause of worn dentition was inappropriate overcontoured restorations of upper anterior teeth (Figure 3). Missing of #6,11,23,26, crown restoration of tooth #9, and two fixed partial dentitions (FPDs) to replace teeth #6 and 11 were evident. Tooth number #30 had old endodontic treatment with direct amalgam filling (Figure 4). Bilateral lingual torus mandibularis in canine/premolar positions were observed. VDO was evaluated and the freeway space was measured to be 5 mm (normal value is 2–4 mm).^10^

The possible treatment planes included increasing VDO by surgical, orthodontic, or restorative treatments to provide enough restorative space for anterior teeth restorations. The patient reported unsuccessful experience by previous orthodontic treatment (6 years ago) and rejected two first options. Since the posterior teeth were sound and intact, occlusal overlay restorations were considered for reconstruction of posterior teeth to regain the required vertical space in the most conservative approach.

For the diagnostic stage, impressions (Alginate, chromogel) were made and poured by plaster (Moldano Dental Stone, Bayer Co). Centric relation was recorded by bimanual manipulation technique using acrylic anterior deprogrammer (Pattern Resin LS, GC Dental Corp) and bite registration silicone (Futar D; Kettenbach GmbH & Co). The record was used for mounting the primary casts in a semi‐adjustable articulator (Dentatus ARH‐Type; Dentatus AB) by an arbitrary facebow (Dentatus Facebow; Dentatus AB). Since the least amount of VDO opening that satisfy esthetic and functional goals is the VDO of choice, 2 mm increase in anterior segment (1 mm in posterior) was considered.^11^ Wax‐up for lower anterior teeth was carried out after determination of mandibular canines level at the corner of the resting lips, followed by upper anterior teeth waxing. The quality and correctness of waxing were verified by mock‐up in the mouth during phonetics, smiling, and rest position. Finally, the approximate posterior occlusal plane was determined using a Broadrick occlusal plane analyzer. Based on the occlusal plane, it was decided to restore mandibular posterior teeth with ceramic overlay (lithium disilicate, IPS e.max Press, Ivoclar‐Vivadent). Full coverage restorations (porcelain fused to zirconia) were considered for teeth # 9, 24, and 25, teeth # 8,22,27 were reconstructed by lithium disilicate laminates (IPS e.max Press, Ivoclar‐Vivadent), and the missing maxillary teeth were replaced using two fixed prosthetic dentures (FPDs) (porcelain fused to zirconia).

Once the treatment plan was accepted, provisional restorations were made using composite resin (Supreme 3M‐ESPE). The patient used the provisional restorations for 5 months to evaluate mastication ability, muscles comfort, and any complication related to increased VDO. No complication was reported, and temporary restorations were approved. Teeth preparations were completed considering circumferential radial shoulder finish line for FPDs and crowns, and light chamfer for laminates. Light anatomic preparation without peripheral reduction of occlusal surface of mandibular posterior teeth was performed in this step. The reason for selecting anatomic preparation design is because of desirable fracture resistance and this design decrease the amount of enamel reduction.^12^ The final impressions were taken by 2 consistency‐2 phase impression technique using putty and extra‐light body impression materials (Panasil, Kettenbach GmbH & Co). The casts were mounted by cross mount technique, and final restorations were made based on the silicone index obtained from provisional restorations and anterior guide table adjusted in provisional step.

**FIGURE 5:**
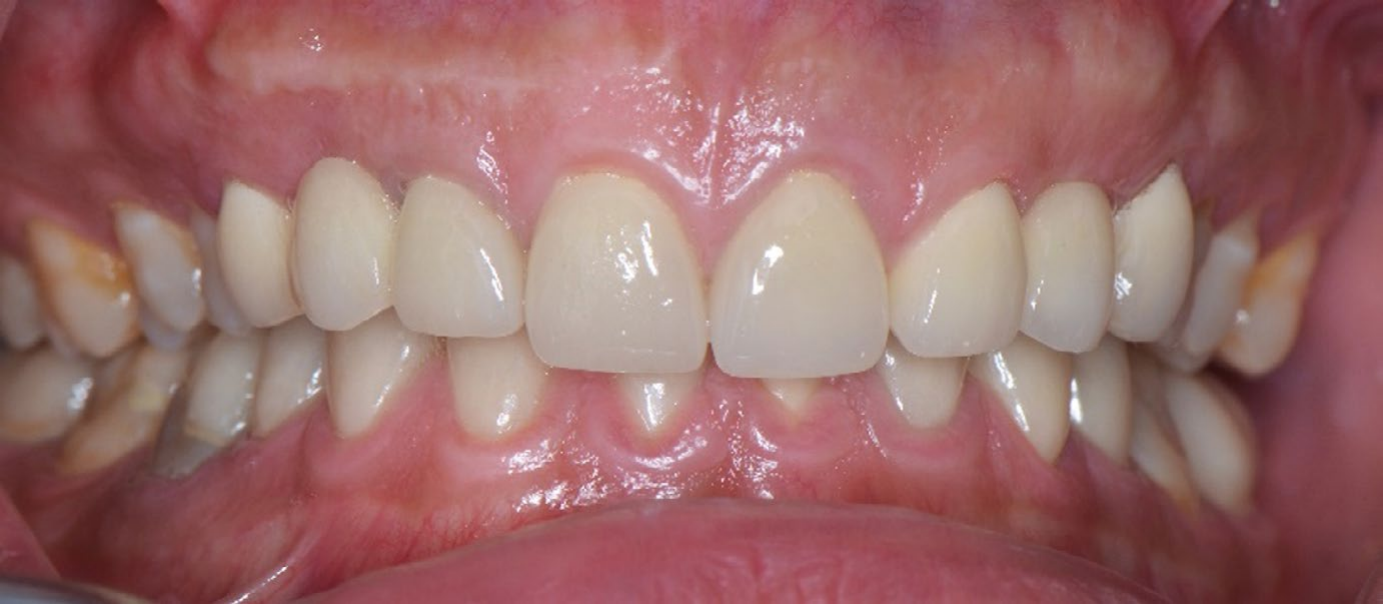
Intraoral frontal view after cementation

**FIGURE 6:**
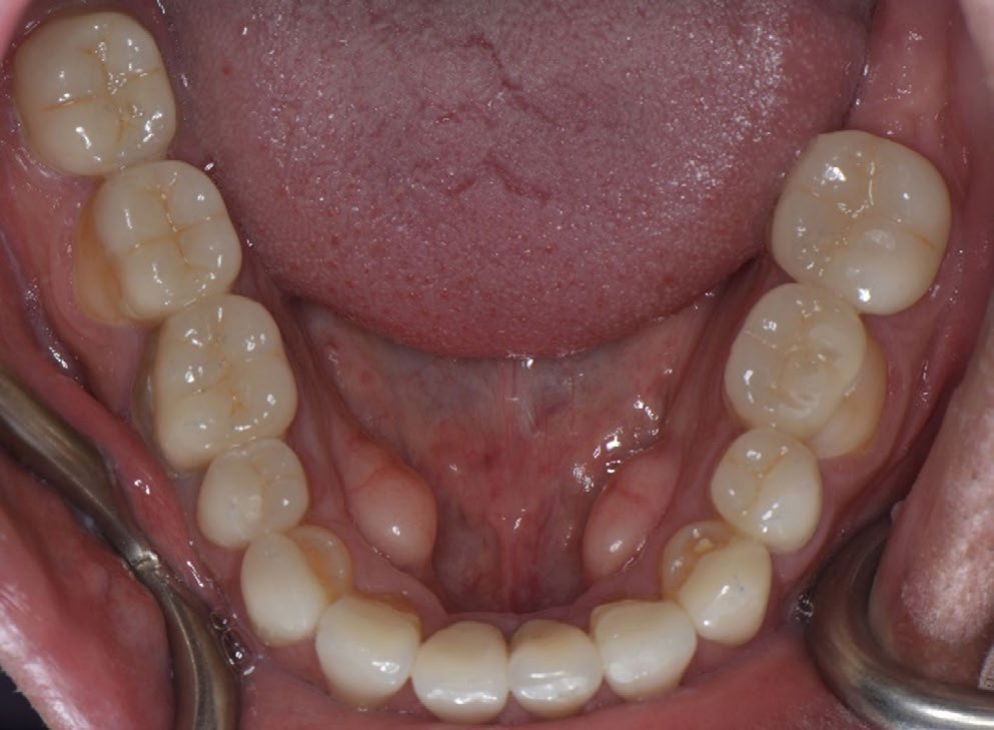
Mandibular occlusal view after cementation

**FIGURE 7:**
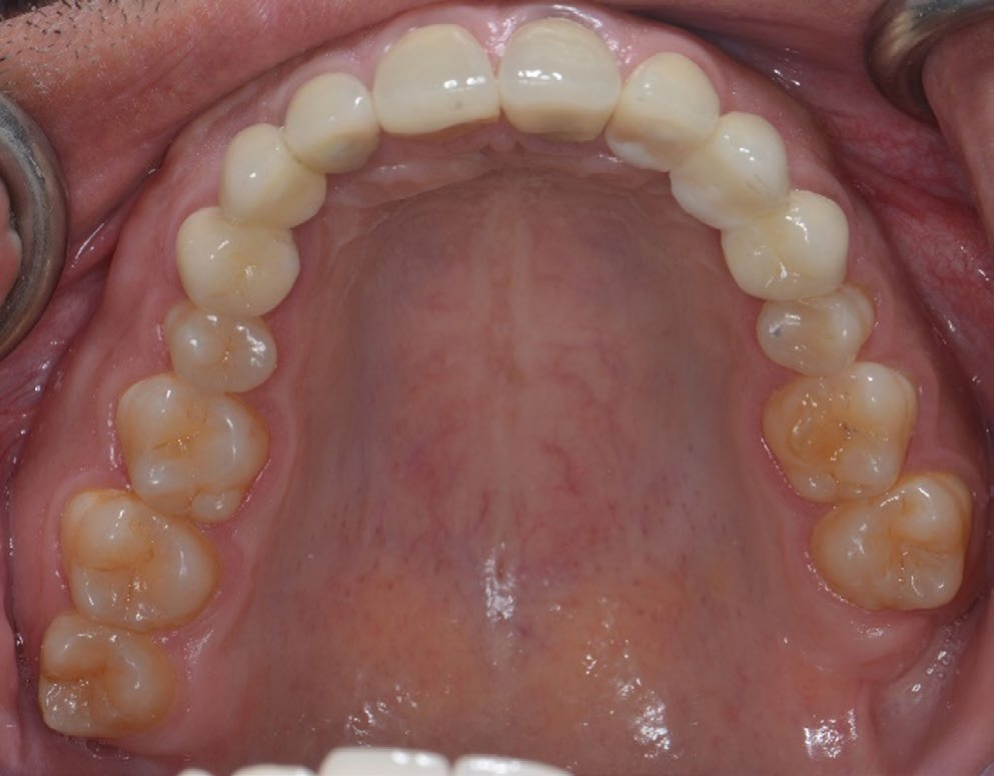
Maxillary occlusal view after cementation

In try‐in session, occlusal contacts were assessed during CR and excursive movements, and group function occlusion was established bilaterally with keeping first premolars and canines in occlusion in working movement. In the delivery session, the occlusal veneers and laminates were cemented by dual‐cure resin cement (Panavia V5, Kuraray Co), and glass ionomer cement (Fuji II, GC Dental Corp) was used for cementing full coverage restorations (Figures 5, 6, 7). The patient's chief complaints were effectively resolved, and the patient was very satisfied with the functional and aesthetic outcomes. Oral hygiene instructions, using water jet, and super floss was explained for the patient, and follow‐up sessions were set for 1, 6, and 12 months later, and annually afterward. At a review appointment 1 month later, the patient reported a clicking sound in opening; therefore, a dual stabilization appliance was fabricated for patient and the next appointment was set 2 weeks later. After 3 years, no evidence of bone loss or loss of VDO, and no signs of TMD were noted. The restorations had no signs of chipping or wear, and all the surfaces were smooth.

## 3 DISCUSSION

The present case report focused on the treatment procedure of a patient suffered from pathologic deep bite and progressive wear. The challenges were anterior deep bite, severe worn anterior teeth, and the need for increasing VDO in a conservative and durable way.

Pathologic deep bite has some sign and symptoms (including palatal soft tissues trauma, unstable occlusion, tooth wear, and aesthetic problems) that may necessitate the treatment process. The possible treatment plans might be consisted of reshaping of malformed restorations, orthodontic treatment, orthognathic surgery, and prosthetic reconstruction.^4^, ^13^, ^14^ One of the challenges in deep bite patients is related to available restorative space for needed restorations. The treatment could become even more complicated when the occlusal wear occurs in a deep bite patient. Dento‐alveolar compensation that happens continuously and is accelerated by wear problems, eliminates the space required for reconstruction of worn teeth. Several treatment options could be used in this situation to provide the needed structure for retention, stability, and esthetic.

1. Orthodontic intrusion and proclination of supraerupted teeth.
2. Reduction of teeth in same or opposing arch, occlusal plane adjustment, and occlusal reestablishing with or without surgical crown lengthening.
3. Increasing the VDO on posterior teeth (by Dahl appliance, direct, or indirect full or partial coverage restorations, or orthodontic extrusion of posterior teeth).
4. Elective endodontic treatment to provide enough space and retention from inside of tooth structure using post‐retained restoration or Richmond crown.^4^, ^14^, ^15^, ^16^

Each of these options has its own indications and considerations. On the other hand, the wear of buccal surface of mandibular anterior teeth (the present case) is one of the problems needs increasing VDO in most of the cases.^5^ VDO could be increased with fixed prosthesis or removable appliance. Regarding the method of increasing VDO, studies that used fixed prosthesis reported less complication in comparison with studies that used removable appliance.^15^, ^17^ The Dahl technique uses a localized appliance or restorations in supra‐occlusion position on anterior teeth to let the posterior part of dentition extrudes for full arch contacts over a period of time.^15^ However, the success of this appliance completely depends on patient cooperation and several complications have been reported during treatment including possible TMJ dysfunction and tenderness, unpredictable tooth movement, and tilt or drift of other teeth.^14^ Another method for increasing VDO, orthodontic extrusion, usually involves posterior extrusion of teeth combined with anterior‐posterior repositioning of anterior teeth with limited intrusion. This is while intrusion is considerably more complex with adults and orthodontic treatment requires 6–12 months, and it is important to equalize the mesiodistal spacing that occurs as the teeth are repositioned anteriorly.^18^ Another more prevalent treatment option to regain restorative space in these patients is increasing VDO by restorative treatment of posterior teeth using direct composite resin, or indirect full‐ or partial coverage restorations. Partial coverage ceramic overlay seems to be the most conservative durable esthetic option among mentioned restorative methods. However, choosing particular appropriate preparation design and ceramic material play an important role in long‐term success of this treatment.^5^, ^19^

Several factors including the amount of residual tooth structure, types of restoration material, finish line design, thickness of restorations, types of cement could affect the mechanical behavior of occlusal veneers.^19^, ^20^, ^21^

There are several ceramic materials available to be potentially used in low thickness for high‐stress occlusal veneers. High‐glass ceramics have less compressive strength, while low‐glass types like lithium disilicate (LDS), zirconia reinforced lithium silicate, and hybrid ceramics provide suitable esthetic and increasingly higher strength. The bonding of glass ceramics is an important feature for application in partial coverage restorations.^22^

LDS glass ceramic, with acceptable scientific support for application, is a durable high esthetic material with a flexural strength of 360 MPa that can be used for reconstruction of worn teeth as partial or full coverage restorations.^23^, ^24^, ^25^ The minimum clinically acceptable thickness for LDS occlusal veneer restorations is 0.6 mm.^19^, ^25^ Clausen et al. reported higher fracture resistance of occlusal veneers bonded to enamel (in comparison with dentin), and lithium disilicate glass ceramic (compare to leucite reinforced ceramic). No significant difference was reported for different finish line designs (straight‐beveled or chamfer finishing line). ^26^ Angerame et al. reported no significant difference between different preparation designs (rounded shoulder /marginal chamfer) regarding fracture resistance and marginal adaptation of occlusal veneer restorations (IPS e.max CAD). ^27^

Polycrystalline no‐glass ceramics like zirconia have the highest mechanical strength (flexural strength of more than 900 MPa, hardness of 1200–1350 HVN, and fracture toughness of 6–15 MPa m^0.5^) and provide conservative preparation (0.5 mm thickness shows suitable clinical outcome) and acceptable aesthetic in translucent types.^22^, ^23^, ^28^, ^29^ However, challenges in their bonding ability, put a big question mark against their routine application in partial coverage restoration.^22^, ^24^

Zirconia‐reinforced lithium silicate (ZLS), Celtra DuoTM (Dentsply, Degudent), and VITA Suprinity^®^, (VITA Zahnfabrik), is a glass ceramic material enriched with zirconia (≈10% by weight) that shows flexural strength of 443.63 MPa and fracture toughness 2.31 MPa m^0.5^.^23^ Studies confirm that this material in 0.6 mm thickness can be used for reconstruction of posterior worn teeth.^19^, ^30^

Polymer infiltrated ceramic network materials (VITA Enamic) which consisting two parts (interpenetrate of a methacrylate polymer into a porous ceramic network) have been developed as a CAD/CAM material. Based on evidence, this material has both advantages of composite resin and ceramics, alleviate procedure of milling with flexural resistance of 150–160 MPa and thickness of 0.6 mm and considered as an acceptable one for reconstruction of worn teeth.^24^, ^29^ CAD/CAM resin Nano Ceramics (Lava Ultimate) with flexural resistance of 200 MPa and CAD/CAM composite (3M Paradigm MZ100) with 150 MPa flexural resistance in 0.6 mm‐ thickness are considered as two reliable materials in reconstruction of posterior worn teeth.^21^, ^31^

Despite the fact that composite resin, LDS, ZLS, and hybrid ceramics could be used for posterior occlusal veneer, but controversies exist among superiority of ceramic or composite resin restoration for posterior occlusal veneers.^30^, ^31^, ^32^ In current case, LDS occlusal veneers were used for increasing VDO. It appears that this conservative treatment approach is reliable for both purpose of function and aesthetic. More long‐term clinical trials are needed to clarify the best material, preparation design, and thickness for predictive clinical outcomes.

## 4 CONCLUSION

This article presented a case report of a patient affected by worn teeth. Deep bite, over contoured restorations, inadequate restorative space were some challenges in this case. Using minimally invasive approach and choosing appropriate material for reconstruction of worn teeth were noticed and 3 years follow‐up confirmed patient satisfaction and success in our treatment.

## CONFLICT OF INTEREST

The authors have no conflict of interest in this study.

## AUTHOR CONTRIBUTIONS

MH: prosthodontic treatment of patient, wrote the manuscript. SG: prosthodontic treatment of patient, study conception and design. NY: study conception and design, and wrote the manuscript.

## ETHICAL APPROVAL

Appropriate informed consent was taken for publication of this report and the associated images and collected in accordance with the journal's patient consent policy.

## CONSENT

Published with written consent of the patient.

## Data Availability

The data used to support the findings of this study are available from the corresponding author upon request.
